# Supplementary material for: The Systems Biology Research Tool: evolvable open-source software
Source: BMC Syst Biol. 2008 Jun 29;2:55. doi: 10.1186/1752-0509-2-55 (PMC2446383; doi:10.1186/1752-0509-2-55)
Supplement: Additional file 1 — SBRT Archive. An archive of the current version of the Systems Biology Research Tool. [file 1752-0509-2-55-S1.zip › sbrt-1.4.0/doc/developers_guide/api/index-files/index-13.html]

M-Index


|  |  |  |
| --- | --- | --- |
| **Overview** \| Package \| Class \| Use \| **Tree** \| **Deprecated** \| **Index** \| **Help** | |  |
| **PREV LETTER**   **NEXT LETTER** | **FRAMES**    **NO FRAMES**     **All Classes** |  |


A B C D E F G H I J K L M N O P Q R S T U V W X Y Z 

---


## **M**

**main(String[])** - Static method in class sbrt.ext.mma.algebra.MathematicaSolver: Used for testing purposes. **main(String[])** - Static method in class sbrt.ext.R.stat.RCorrelator: Used for testing purposes. **main(String[])** - Static method in class sbrt.kernel.fba.AbstractRxn: Used for testing purposes. **main(String[])** - Static method in class sbrt.kernel.math.HashLinearComb: Used for testing purposes. **main(String[])** - Static method in class sbrt.kernel.math.stat.comp.KendallTau: Tests the Kendall Tau A and B computers. **main(String[])** - Static method in class sbrt.kernel.math.stat.comp.MannWhitneyU: Used for testing. **main(String[])** - Static method in class sbrt.kernel.util.Util: Used for testing purposes. **main(String[])** - Static method in class sbrt.Sbrt: Parses the provided command line arguments and executes the application they specify. **main(String[])** - Static method in class sbrt.shell.text.fba.BiggBracketParserV1: Used for testing purposes. **MAJOR** - Static variable in class sbrt.Sbrt: Indicates the major version number of the Systems Biology Research Tool. **make(Collection<String>, IrrevRxnFluxome)** - Static method in class sbrt.kernel.fba.IrrevRxnFluxomeV1: Returns an irreversible reaction fluxome composed of the reactions in the provided fluxome with the names in the provided collection. **make(LinearSystemSolution<V>, Map<? extends V, Interval>, double)** - Static method in class sbrt.kernel.math.geom.HashConvexPolytope: Constructs a new convex poltyope from the provided linear system solution and variable bounds. **make(ManagerInput<String, String>, PrintWriter)** - Static method in class sbrt.shell.ProcessManagerFactory: Prints a formatted string representation of the provided input to the provided print writer and returns its corresponding process manager. **make(ManagerInput<String, String>, PrintWriter)** - Static method in class sbrt.shell.ProgramSolverFactory: Uses the information contained in the provided input to create a new program solver object. **makeFluxome(T)** - Method in interface sbrt.kernel.fba.comp.FluxomeReducer: Returns a fluxome that has been reduced in size. **makeFluxome(IrrevRxnFluxome)** - Method in class sbrt.kernel.fba.comp.MsFluxomeReducer: Returns the sub-fluxome resulting from linear programming. **makeFluxome(IrrevRxnFluxome)** - Method in class sbrt.kernel.fba.comp.WwFluxomeReducer: Returns the sub-fluxome resulting from linear programming. **ManagerInput**<K,V> - Interface in sbrt.shell.mng: This interface is used to represent the input to `KernelProcessManager` objects. **MannWhitneyResults** - Class in sbrt.kernel.math.stat.comp: This class is used to store the results of a Mann-Whitney U test. **MannWhitneyResults(double, double, int, int)** - Constructor for class sbrt.kernel.math.stat.comp.MannWhitneyResults: Constructs a new set of results for a Mann-Whitney U test. **MannWhitneyResults(double, int, int)** - Constructor for class sbrt.kernel.math.stat.comp.MannWhitneyResults: Constructs a new set of results for a Mann-Whitney U test. **MannWhitneyU** - Class in sbrt.kernel.math.stat.comp: This class is used to compute Mann-Whitney U statistics. **MannWhitneyU()** - Constructor for class sbrt.kernel.math.stat.comp.MannWhitneyU: **MannWhitneyUManager** - Class in sbrt.shell.mng.stat: This class is used to manage Mann-Whitney tests. **MannWhitneyUManager()** - Constructor for class sbrt.shell.mng.stat.MannWhitneyUManager: **MapEntryFormat**<K,V> - Interface in sbrt.shell.text: This interface is used to represent formats for map entries. **MapFormat**<K,V> - Interface in sbrt.shell.text: This interface is used to represent formats for maps. **MapInputFile**<K,V> - Interface in sbrt.shell.io: This interface is used to represent input files that contain maps. **MapManagerInput**<K,V> - Class in sbrt.shell.mng: This class is a concrete implemenation of `ManagerInput`. **MapManagerInput(Map<K, V>, String)** - Constructor for class sbrt.shell.mng.MapManagerInput: Constructs a new manager input from the provided map and name. **MapManagerInput(Map<K, V>)** - Constructor for class sbrt.shell.mng.MapManagerInput: Constructs a new manager input from the provided map with a `null` name. **MapOutputFile**<K,V> - Interface in sbrt.shell.io: This interface is used to represent output files that maps can be written to. **MapTextFile**<K,V> - Interface in sbrt.shell.io: This interface is used to represent text-based files that contain maps. **MapTextInputFile**<K,V> - Interface in sbrt.shell.io: This interface is used to represent text-based input files that contain maps. **MapTextOutputFile**<K,V> - Interface in sbrt.shell.io: This interface is used to represent output files that maps can be written to as text. **MathematicaException** - Exception in sbrt.ext.mma: This class is used to wrap exceptions thrown by Mathematica. **MathematicaException(MathLinkException)** - Constructor for exception sbrt.ext.mma.MathematicaException: Constructs an external software exception. **MathematicaException(String, MathLinkException)** - Constructor for exception sbrt.ext.mma.MathematicaException: Constructs an external software exception. **MathematicaSolver**<V> - Class in sbrt.ext.mma.algebra: This class is used to solve systems of linear equations using Mathematica. **MathematicaSolver(String[])** - Constructor for class sbrt.ext.mma.algebra.MathematicaSolver: Creates a new linear system solver. **MathematicaSolver(String)** - Constructor for class sbrt.ext.mma.algebra.MathematicaSolver: Creates a new linear system solver. **MathExpr**<V> - Interface in sbrt.kernel.math: This interface is used to represent mathematical expressions. **MathExprFormat**<E extends MathExpr<V>,V> - Interface in sbrt.shell.text.math: This interface is used to represent formats for linear combinations. **Matrix**<R,C,E> - Interface in sbrt.kernel.math.algebra: This interface is used to represent matrices. **MATRIX\_FILE\_NAME** - Static variable in class sbrt.shell.mng.fba.NetworkInfoManager: The keyword used to denote the name of the file to which the stoichiometry matrix of a fluxome will be written. **MATRIX\_FILE\_NAMES\_FILE** - Static variable in class sbrt.shell.mng.fba.StoichVariationManager: The keyword used to denote the name of the file containing the names of the stiociometry matrix variation files. **MatrixBuilder**<R,C,E> - Interface in sbrt.kernel.math.algebra: This interface is used to represent mutable matrices. **MatrixConverterManager** - Class in sbrt.shell.mng.algebra: This class is used to convert matrices to linear combinations. **MatrixConverterManager()** - Constructor for class sbrt.shell.mng.algebra.MatrixConverterManager: **MatrixElement**<R,C,E> - Class in sbrt.kernel.math.algebra: This class is used to represent matrix elements. **MatrixElement(S, T, U)** - Constructor for class sbrt.kernel.math.algebra.MatrixElement: Constructs a new matrix element. **MatrixElement(IndexPair<R, C>, U)** - Constructor for class sbrt.kernel.math.algebra.MatrixElement: Constructs a new matrix element. **MatrixElementFormat**<R,C,E> - Interface in sbrt.shell.text.math: This interface is used to represent formats for matrix elements. **MatrixElementFormatV1**<R,C> - Class in sbrt.shell.text.math: This class is a concrete implementation of `MatrixElementFormat`. **MatrixElementFormatV1(SimpleFormat<R>, SimpleFormat<C>)** - Constructor for class sbrt.shell.text.math.MatrixElementFormatV1: Constructs a new matrix element format. **MatrixMultiplier** - Class in sbrt.kernel.math.algebra: This class is used to multiply matrices. **MatrixMultiplier()** - Constructor for class sbrt.kernel.math.algebra.MatrixMultiplier: **MatrixTransposer** - Class in sbrt.kernel.math.algebra: This class is used to transpose matrices. **MatrixTransposer()** - Constructor for class sbrt.kernel.math.algebra.MatrixTransposer: **MatrixWrap**<R,C,E> - Class in sbrt.kernel.math.algebra: This class is used to wrap mutable matrices as an immutable matrix. **MatrixWrap(Matrix<R, C, E>)** - Constructor for class sbrt.kernel.math.algebra.MatrixWrap: Constructs a new wrapper around the provided matrix. **MAX\_OBSTRUCTED\_MOVES** - Static variable in class sbrt.shell.mng.geom.CdHarManager: The keyword used to denote the maximum number of obstructed moves to allow. **MetatoolFileWriter** - Class in sbrt.shell.proc.fba: This class is used to write input files for Metatool. **MetatoolFileWriter()** - Constructor for class sbrt.shell.proc.fba.MetatoolFileWriter: **MetatoolFileWriterManager** - Class in sbrt.shell.mng.fba: This class is used to manage the writing of input files for Metatool. **MetatoolFileWriterManager()** - Constructor for class sbrt.shell.mng.fba.MetatoolFileWriterManager: **MIN\_CHORD\_LENGTH** - Static variable in class sbrt.shell.mng.geom.CdHarManager: The keyword used to denote the minimum chord length. **MINOR** - Static variable in class sbrt.Sbrt: Indicates the minor version number of the Systems Biology Research Tool. **MissingVariableException** - Exception in sbrt.kernel.math: This class is used to indicate that a varible is missing. **MissingVariableException(Object)** - Constructor for exception sbrt.kernel.math.MissingVariableException: Constructs an exception to indicate the provided variable is missing. **MMA\_NOTEBOOK\_NAME** - Static variable in class sbrt.shell.mng.fba.MmaNotebookManager: The keyword used to denote the name of the Mathematica notebook to be written. **MMaExprInterpretter** - Class in sbrt.ext.mma.algebra: This class is intended for use solely by the class `MathematicaSolver`. **MmaNotebookManager** - Class in sbrt.shell.mng.fba: **MmaNotebookManager()** - Constructor for class sbrt.shell.mng.fba.MmaNotebookManager: **MonitoredProcess**<R> - Interface in sbrt.kernel: This interface is used to represent monitored processes. **MsFluxIntervalComputer** - Class in sbrt.kernel.fba.comp: This class is used to compute Mahadevan-Schilling flux intervals. **MsFluxIntervalComputer(FbaOptComputer, Map<String, Set<LinearComb<String>>>)** - Constructor for class sbrt.kernel.fba.comp.MsFluxIntervalComputer: Constructs a new Mahadevan-Schilling flux interval computer. **MsFluxIntervalComputer(FbaOptComputer, Map<String, Set<LinearComb<String>>>, double)** - Constructor for class sbrt.kernel.fba.comp.MsFluxIntervalComputer: Constructs a new Mahadevan-Schilling flux interval computer. **MsFluxIntervalManager** - Class in sbrt.shell.mng.fba: This class is used to manage Mahadevan-Schilling flux interval experiments. **MsFluxIntervalManager()** - Constructor for class sbrt.shell.mng.fba.MsFluxIntervalManager: **MsFluxomeReducer** - Class in sbrt.kernel.fba.comp: This class is used to reduce the size of fluxomes for the purpose of identifying the steady state cycles (Type III extreme pathways) they contain. **MsFluxomeReducer(ProgramSolver<Object, Object>)** - Constructor for class sbrt.kernel.fba.comp.MsFluxomeReducer: Constructs a fluxome reducer from the provided linear program solver. **MsFluxomeReducerManager** - Class in sbrt.shell.mng.fba: This class is used to manage the MS fluxome reduction algorithm. **MsFluxomeReducerManager()** - Constructor for class sbrt.shell.mng.fba.MsFluxomeReducerManager: **MultiOutputFiles**<F extends OutputFile<R>,R> - Class in sbrt.shell.io: This class is used to create multiple output files. **MultiOutputFiles(Iterator<F>)** - Constructor for class sbrt.shell.io.MultiOutputFiles: Constructs a new multiple output files results manager. **MultipleCorrelationAnalysis**<V,T extends CorrResult> - Class in sbrt.kernel.math.stat.proc: This class is used to perform multiple correlation analyses. **MultipleCorrelationAnalysis(Correlator<V, T>)** - Constructor for class sbrt.kernel.math.stat.proc.MultipleCorrelationAnalysis: Constructs a new correlation experiment. **MultipleVectorsConverterManager** - Class in sbrt.shell.mng.algebra: This class is used to convert files containing multiple vectors into a series of files, each containing a single vector. **MultipleVectorsConverterManager()** - Constructor for class sbrt.shell.mng.algebra.MultipleVectorsConverterManager: **multiply(SparseDoubleMatrix<R, C>, SparseDoubleMatrix<C, T>, DoubleMatrixBuilder<R, T>)** - Method in class sbrt.kernel.math.algebra.MatrixMultiplier: Fills `product` with `matrixA` \* `matrixB`. **multiply(double)** - Method in class sbrt.kernel.math.HashLinearComb: Multiplies this linear combination by the provided constant. **multiply(double)** - Method in interface sbrt.kernel.math.LinearComb: Multiplies this linear combination by the provided constant. **MutableProgressMonitor** - Interface in sbrt.kernel.util: This interface is used to represent mutable progress montiors. **MwUAnalysis**<V> - Class in sbrt.kernel.math.stat.proc: This class is used to perform Mann-Whitney U tests. **MwUAnalysis()** - Constructor for class sbrt.kernel.math.stat.proc.MwUAnalysis: Constructs a new Mann-Whitney U analysis.

---


|  |  |  |
| --- | --- | --- |
| **Overview** \| Package \| Class \| Use \| **Tree** \| **Deprecated** \| **Index** \| **Help** | |  |
| **PREV LETTER**   **NEXT LETTER** | **FRAMES**    **NO FRAMES**     **All Classes** |  |


A B C D E F G H I J K L M N O P Q R S T U V W X Y Z 

---
